# Dioxin (TCDD) Induces Epigenetic Transgenerational Inheritance of Adult Onset Disease and Sperm Epimutations

**DOI:** 10.1371/journal.pone.0046249

**Published:** 2012-09-26

**Authors:** Mohan Manikkam, Rebecca Tracey, Carlos Guerrero-Bosagna, Michael K. Skinner

**Affiliations:** Center for Reproductive Biology, School of Biological Sciences, Washington State University, Pullman, Washington, United States of America; Massachusetts General Hospital, United States of America

## Abstract

Environmental compounds can promote epigenetic transgenerational inheritance of adult-onset disease in subsequent generations following ancestral exposure during fetal gonadal sex determination. The current study examined the ability of dioxin (2,3,7,8-tetrachlorodibenzo[p]dioxin, TCDD) to promote epigenetic transgenerational inheritance of disease and DNA methylation epimutations in sperm**.** Gestating F0 generation females were exposed to dioxin during fetal day 8 to 14 and adult-onset disease was evaluated in F1 and F3 generation rats. The incidences of total disease and multiple disease increased in F1 and F3 generations. Prostate disease, ovarian primordial follicle loss and polycystic ovary disease were increased in F1 generation dioxin lineage. Kidney disease in males, pubertal abnormalities in females, ovarian primordial follicle loss and polycystic ovary disease were increased in F3 generation dioxin lineage animals. Analysis of the F3 generation sperm epigenome identified 50 differentially DNA methylated regions (DMR) in gene promoters. These DMR provide potential epigenetic biomarkers for transgenerational disease and ancestral environmental exposures. Observations demonstrate dioxin exposure of a gestating female promotes epigenetic transgenerational inheritance of adult onset disease and sperm epimutations.

## Introduction

Epigenetic transgenerational inheritance involves the germline transmission of an altered epigenome and phenotypes across generations in the absence of direct environmental exposures [1], [2]. The germline epigenome undergoes reprogramming during fetal gonadal development [3]. Environmentally induced germline epigenetic modifications can occur during this DNA demethylation and remethylation period [1] and become permanently programmed similar to the DNA methylation of an imprinted gene [4]. The male germline propagates this epigenetic change after fertilization to all somatic cells resulting in an altered epigenome and transcriptome that can lead to adult onset disease in future generations. A number of environmental chemical exposures have been shown to promote epigenetic transgenerational inheritance of adult onset disease and the transgenerational epigenetic changes may be used as biomarkers of exposure and disease [5].

The current study was designed to investigate the potential that dioxin (2,3,7,8-tetrachlorodibenzo[p]dioxin, TCDD) promotes epigenetic transgenerational inheritance of adult onset disease. In rodents TCDD has a half-life of weeks and causes liver disease, weight loss, thymic atrophy and immune suppression. In humans direct dioxin exposure influences chronic diseases, lymphomas and leukemias [6]. The half-life of TCDD in humans varies to over 10 years with body mass index, age, sex and exposure concentration [7]. Agent Orange is one of the TCDD-contaminated herbicides used by the U.S. military during the Vietnam War from 1961 to 1971. Vietnam officials estimate 400,000 people were killed or maimed and 500,000 children born with birth defects resulting from exposure to Agent Orange [8]. The diseases associated with exposure to Agent Orange include: prostate cancer, respiratory cancers, multiple myeloma, type II diabetes, Hodgkin's disease, non-Hodgkin's lymphoma, soft tissue sarcoma, chloracne, porphyria cutanea tarda, peripheral neuropathy, chronic lymphocytic leukemia, spina bifida in children, B cell leukemias (such as hairy cell leukemia), Parkinson's disease and ischemic heart disease [7]. Another example of a major human exposure to TCDD was the Anshu Seveso Italy industrial accident that occurred in 1976 [9]. Human exposure to dioxin from electronic waste in China has also been documented [10]. A Taiwan industrial accident and food contamination in 1979 was another major incidence of human exposure [11]. Therefore, a number of different human exposures to dioxin have been documented and associated with a large variety of different disease states. The majority of epidemiology studies have focused on direct adult and fetal exposures [12]. A study of the Seveso Italy population documented health effects in the grandchildren (F2 generation) of women that conceived as long as 25 years after the dioxin exposure [13]. No human studies have investigated transgenerational (F3 generation) effects of dioxin.

Animal models have been used to study the toxicological effects of dioxin. Dioxin has been shown to produce cleft palates and kidney malformations in newborn mice [14]. Adverse effects in animals include endometriosis, developmental neurobehavioral (cognitive) effects, developmental reproductive (sperm counts, female urogenital malformations) effects and immunotoxic effects [15]. A study on pregnant mice exposed to dioxin showed 50% pup mortality [16]. Previous studies with dioxin used high doses (0.2 to 3 µg/kg/BW) and only evaluated the direct exposure of adult and fetal (F0 and F1) generations [17], [18]. The current study used 0.1% of oral LD50 dose for TCDD, such that no toxic effects of the exposure were anticipated. However, the current study was not designed as a risk assessment study, but to investigate the potential that dioxin may promote transgenerational disease. Since the exposure of a gestating F0 generation female also directly exposes the F1 generation fetus and germ line that will generate the F2 generation, the current study investigated the F3 generation which is the first generation without direct exposure [19].

Environmental chemicals (fungicide vinclozolin and pesticide methoxychlor) were initially found to promote epigenetic transgenerational inheritance of adult onset diseases following ancestral exposure [2]. Exposure of gestating females transiently to vinclozolin during the fetal gonadal sex determination period promoted epigenetic transgenerational inheritance of adult onset diseases in the F1-F4 generation rats. Subsequently a variety of environmental chemicals have been shown to promote epigenetic transgenerational inheritance of adult onset disease including the plasticizers bisphenol A (BPA) [5], [20], [21], [22] and phthalates [5], [20], [22], pesticide permethrin and insect repellent DEET [5], [22], [23], and hydrocarbon mixture (jet fuel JP8) [5], [22], [24]. A number of other environmental factors such as nutrition (caloric restriction) have also been shown to promote transgenerational phenotypes [25]. Epigenetic transgenerational inheritance has now been shown to be present in plants [26], worms [27], flies [28], rats [5], mice [29] and humans [30]. The first observation that dioxin promotes transgenerational inheritance of adult onset disease demonstrated a decline in fertility in the F3 generation following dioxin exposure to F0 generation gestating female mice [31]. Subsequently, dioxin exposure was found to promote pubertal abnormalities and ovarian disease in 120 day old F3 generation rats [5]. The current study was designed to extend these observations with dioxin to examine the epigenetic transgenerational inheritance of a variety of different disease states in 1 year old F1 (direct exposure) and F3 (transgenerational) generation rats.

The epigenetic mechanisms involved in the transgenerational inheritance of disease have been previously reviewed [1]. Exposure of the fetus during gonadal sex determination alters the epigenetic (DNA methylation) programming of the germ line (e.g. sperm) that then transmits this altered epigenome in an imprinted-like manner between generations to promote adult onset disease transgenerationally [4]. These sperm epimutations in the F3 generation dioxin lineage are unique and may be useful as biomarkers of dioxin exposure and adult-onset disease [5]. The current study further investigates the dioxin induced epimutations associated with the sperm epigenome.

## Results

The epigenetic transgenerational action of dioxin administered to F0 generation female rats transiently during days 8 to 14 of gestation was investigated. The F1 generation animals were bred to generate the F2 generation and the F2 generation bred to generate the F3 generation as previously described [5]. No sibling or cousin breedings were used to avoid any inbreeding artifacts. Control (vehicle dimethysulfoxide DMSO) exposure lineages and dioxin (TCDD) lineages were generated. The objective was to assess the potential transgenerational phenotype so the F3 generation was the focus with comparisons with the direct exposure F1 generation, while the F2 generation was not examined. The F1 and F3 generation rats of control and dioxin lineages were euthanized at 1 year of age. The testis, prostate, kidney and ovary histopathology was examined. To assess if there were any toxic effects from embryonic exposure to dioxin both the F1 and F3 generation body weights and organ weights were measured (Tables S1A and S1B). The body weight of the F1 generation dioxin lineage females was reduced, but the kidney, ovarian and uterine weights were unaltered. The body weight and the epididymal weight did not change in the F1 generation males. Testis weight in the F1 generation males was increased while the prostate and kidney weights were reduced. In the F3 generation dioxin lineage females the body weight, ovarian and uterine weights were unaltered but the kidney weight was reduced. Testis, epididymis and prostate weights did not change, but the kidney weight was lower in the F3 generation males of dioxin lineage. No effect on sex ratios in the F1, F2 or F3 generations were observed. No significant change in litter size were observed. In addition, serum sex steroid hormone concentrations were measured in the F3 generation to assess any endocrine alterations. Serum testosterone concentrations in the 1 year old F3 generation males increased. Serum estradiol concentrations in F3 generation females during proestrus-estrus phase or diestrus phase (Figure S1) were unaltered so no female F3 generation endocrine effects were detected. Observations indicate that there were no major F1 generation toxicological effects from the direct dioxin exposure.

**Figure 1:**
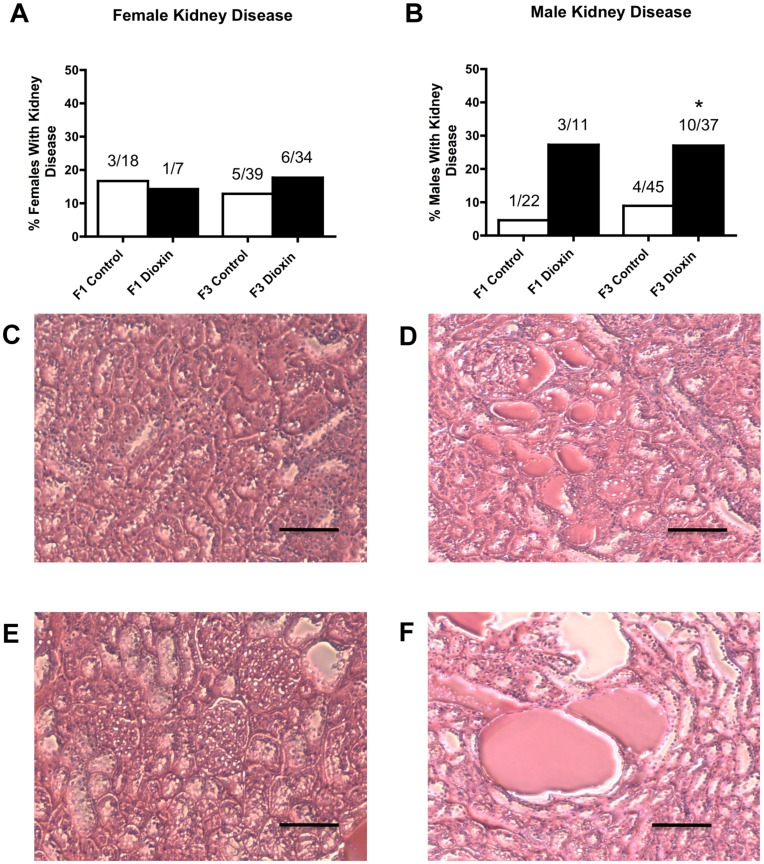
Dioxin and control lineage F1 and F3 generation adult-onset kidney disease. Percentages of females (panel A) and males (panel B) with kidney disease and number of diseased rats/total number of rats (*P<0.05). Micrographs (Scale bar = 200 µm) show kidney disease in F3 generation dioxin lineage (panel E and F) compared to control (panel C and D) for female (panel C and E) and male (panel D and F).

One of the major diseases/abnormalities observed in dioxin lineage males was kidney disease. Kidney disease was characterized by the presence of an increased number of proteinaceous fluid filled cysts, reduction in size of glomeruli and thickening of Bowman’s capsules (Figure 1). Previously, transgenerational kidney phenotypes have been shown to correlate with alterations in serum markers for kidney disease [32]. In the F1 generation an increase in kidney disease in males approached significance (P = 0.0672) and in the females there was no effect. There was a statistically significant increase in kidney disease in F3 generation males, but not in females (Figure 1).

**Figure 2:**
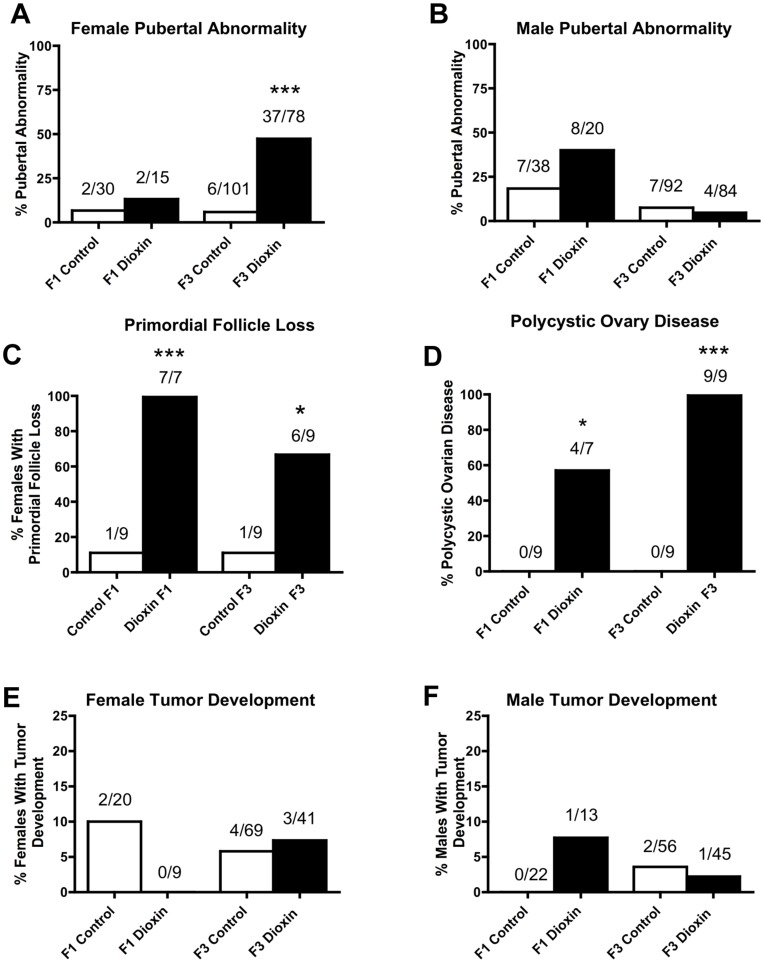
Dioxin and control lineage F1 and F3 generation pubertal abnormality and ovarian disease. Percentages of females (panel A) and males (panel B) with pubertal abnormality, or primordial follicle loss (panel C), or polycystic ovary disease (panel D), or those with tumor development (panels E and F). The number of diseased rats/total number of rats in each lineage are presented (*P<0.05; ***P<0.001).

As previously reported [5], there was an increase in pubertal abnormalities in the F1 generation males of dioxin lineage, but not in F3 generation males (Figure 2). In the F1 generation 40% of males had pubertal abnormalities, with the majority being delayed pubertal onset. In the control lineage 18% of males had pubertal abnormalities, with the majority being delayed pubertal onset. In the F3 generation 5% of dioxin lineage males had pubertal abnormalities, with all of them being delayed onset of puberty. In the F3 generation control lineage 8% of males had pubertal abnormalities, with the majority being early pubertal onset. The incidence of pubertal abnormalities in females did not change in the F1 generation, but was significantly altered in the F3 generation (Figure 2). In the F1 generation 13% of females had pubertal abnormalities, with half being delayed pubertal onset and the other half being early pubertal onset. In control lineage 7% of females had pubertal abnormalities, all being delayed pubertal onset. In the F3 generation 47% of dioxin lineage females had pubertal abnormalities (Figure 2), all being early onset of puberty. In the F3 generation control lineage 6% of females had pubertal abnormalities, with the majority being early pubertal onset.

As previously reported [5], [22] there was an increase in the incidences of ovarian disease/abnormality including primordial follicle loss (Figure 2, panel C) and polycystic ovarian disease (Figure 2, panel D). These data were re-analyzed to determine disease/abnormality incidence for the current study. The primordial follicle loss was shown by a reduction in the number of primordial follicles per ovary section and the polycystic ovarian histopathology was characterized by an increase in the number of small cysts. The F1 and F3 generation females showed an increase in the incidence of both primordial follicle loss and polycystic ovarian disease.

The F1 and F3 generation rats did not present any change in the incidence of tumor development (Figure 2, panels E and F) or incidence of obesity (data not shown). Other sporadic disease predominantly observed in the dioxin lineage animals included abscesses, eye discharges, colon impaction, missing testis, rudimentary epididymis, fat necrosis, lung abnormalities, and active mammary gland (milk presence) unrelated to pregnancy.

**Figure 3:**
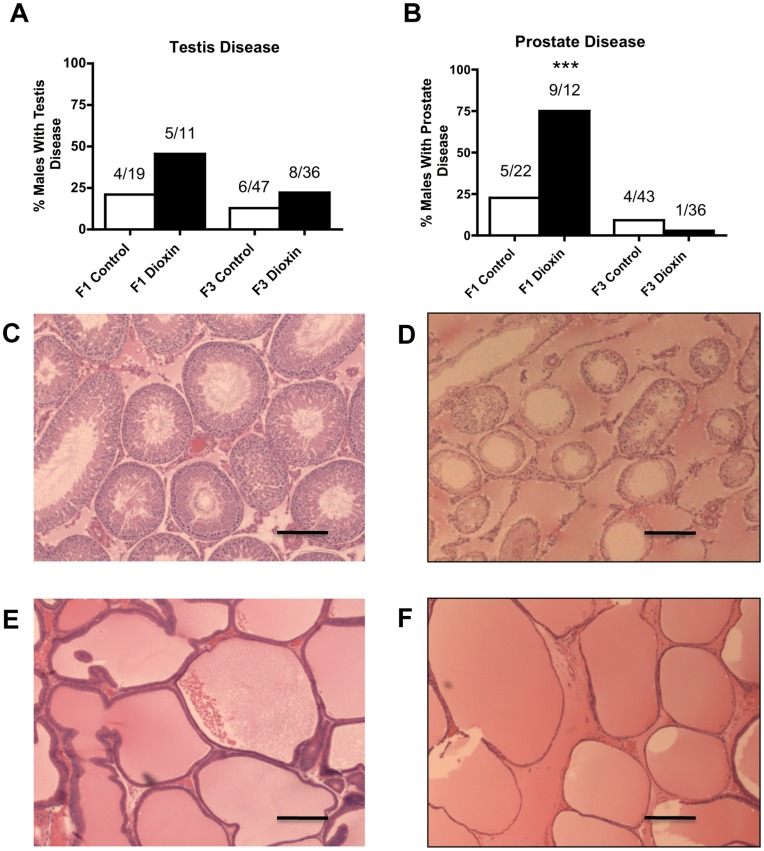
Dioxin and control lineage F1 and F3 generation adult-onset transgenerational testis or prostate disease. Percentages of males with testis (panel A) or prostate disease (panel B) and number of diseased rats/total number of rats (***P<0.001). Micrographs (Scale bar = 200 µm) show testis and prostate disease in F3 generation dioxin lineage (panels D and F) compared to control (panels C and E).

The incidences of testis and prostate diseases in the dioxin lineage are presented in Figure 3. Testis disease/abnormality was characterized by the presence of histopathology including azoospermic and atretic seminiferous tubules, presence of vacuoles in basal regions of seminiferous tubules, sloughed cells in center of seminiferous tubules and lack of seminiferous tubule lumen (Figure 3). There was no increase in testis disease in either F1 or F3 generation rats. To further study testis disease the number of apoptotic spermatogenic cells was examined by TUNEL analysis. The number of apoptotic spermatogenic cells declined in the F1 generation and did not change in the F3 generation rats (Supplemental Figure S1D). Therefore spermatogenic defects that were present in vinclozolin lineage F3 generation rats [2], [32] were not observed in dioxin lineage F3 generation rats. Also there was no alteration in sperm numbers or sperm motility in F1 and F3 generations (data not shown). Prostate disease/abnormality was characterized by atrophic prostatic duct epithelium (Figure 3). Only the F1 generation dioxin lineage rats showed an increase in prostate histopathology.

**Figure 4:**
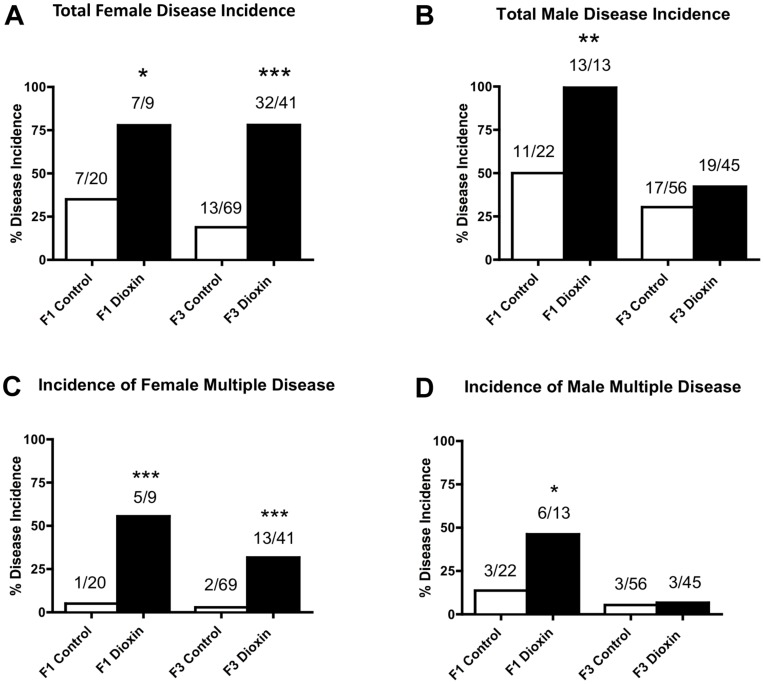
Dioxin and control lineage F1 and F3 generation adult-onset diseases in rats. Incidences of total female disease (panel A), total male disease (panel B), female multiple disease (panel C) and male multiple disease (panel D) and number of diseased rats/total number of rats (*P<0.05; **P<0.01; ***P<0.001).

Observations indicate ancestors exposed to dioxin transgenerationally transmitted kidney disease, pubertal abnormalities and ovarian disease/abnormality to their unexposed F3 generation descendants. These findings are an example of environmentally induced epigenetic transgenerational inheritance of adult-onset disease. The incidence of diseases/abnormalities in individual rats from control and dioxin lineages are presented in Tables S2A (F1 generation females), S2B (F1 generation males), S3A (F3 generation females) and S3B (F3 generation males). The total number of animals is indicated for each disease/abnormality assessment in Tables S2 and S3. The number of animals per litter (litter representation) mean ± SEM used for the control versus dioxin lineage comparison was found not to be statistically different (p>0.05) for each individual disease/abnormality, such that no litter bias was detected. The incidence of total disease/abnormality per rat increased in both F1 and F3 generation females of dioxin lineage (Figure 4, panel A). The incidence of rats with multiple diseases/abnormalities also increased in both F1 and F3 generation females of dioxin lineage (Figure 4, panel C). The incidence of total disease/abnormality per rat increased in F1 and in F3 generation males (Figure 4, panel B). The incidence of multiple diseases/abnormalities per rat increased in F1 generation but not in F3 generation males. Exposure of F0 generation females to dioxin increased the overall incidence of adult onset histopathology in both F1 and F3 generation males and females.

**Figure 5:**
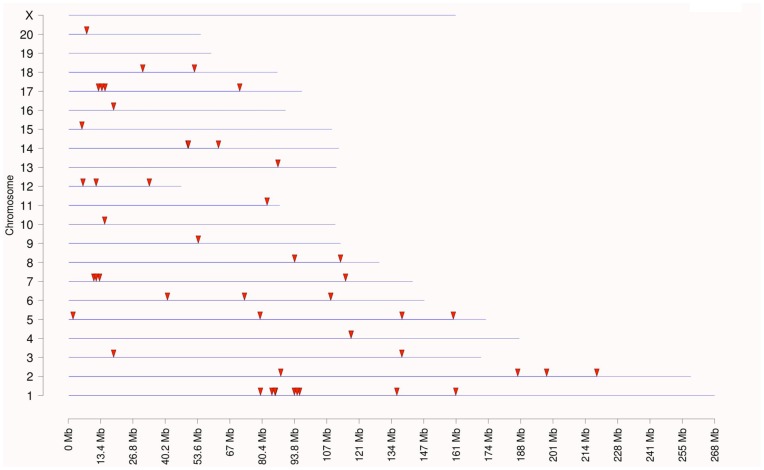
Dioxin promoted F3 generation sperm epimutations. Chromosomal locations for transgenerational differential DNA methylation regions (DMR) (arrowheads). There were 50 DMR in sperm DNA from dioxin lineage rats compared to control lineage rats.

**Figure 6:**
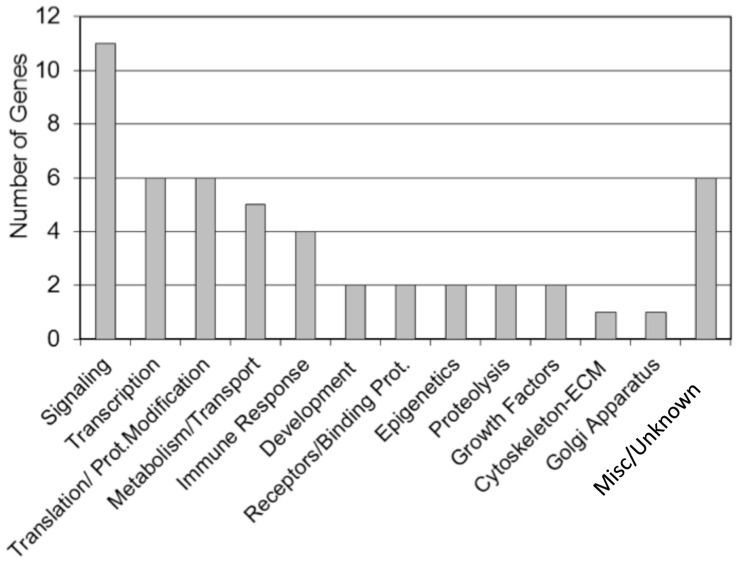
Dioxin induced DMR and functional gene categories. Number of DMR associated with various gene categories.

**Figure 7:**
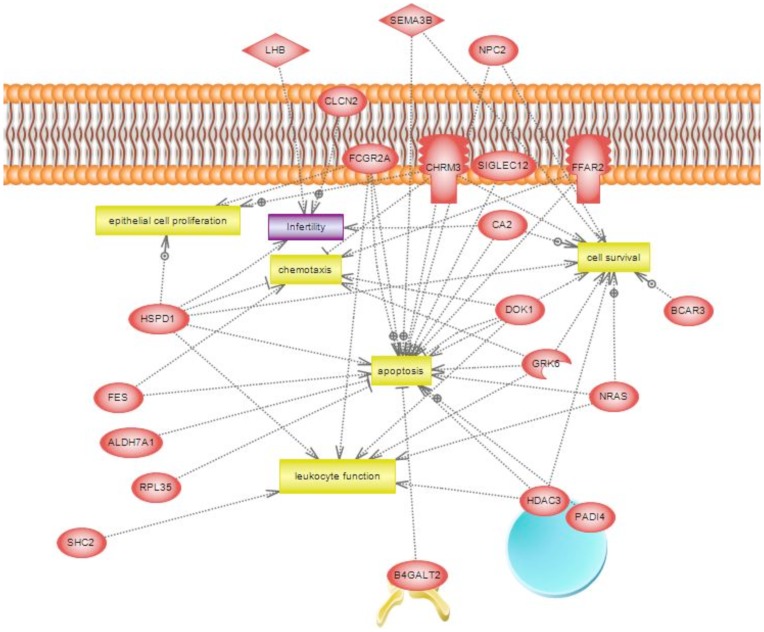
Gene network of DMR associated genes. The DMR associated genes with connections to various cellular processes and associated cellular localization.

As previously described, the transgenerational effects of dioxin on the sperm epigenome are unique [5]. The transgenerational F3 generation control and dioxin lineage sperm epigenomes were analyzed. This analysis identified 50 statistically significant differentially DNA methylated regions (DMR) in promoters, Table 1. A methylated DNA immunoprecipitation (MeDIP) followed by PCR was used to confirm the MeDIP-Chip analysis for selected DMR due to their high connectivity in the gene network below. The MeDIP-PCR for *Hdac3* and *Npc2* were found to confirm the MeDIP-Chip identification with >10 fold changes that were statistically different (p<0.0001). The DMR are on average 800 bp in size. The chromosomal locations of these DMR are presented in Figure 5. The majority of the autosomes contained dioxin induced epimutations. The functional gene categories of the gene promoters containing the DMR are shown in Figure 6. Signaling and transcription were the two most predominant functional gene categories. Signaling pathway and cellular process enrichment for the list of dioxin lineage genes having DMR in F3 generation sperm was examined. The top 20 pathways enriched in genes having DMR in their promoters are presented (Table S4). There were four pathways each with three genes affected. They include ribosome pathway, chemokine signaling pathway and natural killer cell mediated cytotoxicity. Additionally 16 pathways had two genes affected in each. Therefore, dioxin induced a transgenerational alteration in the sperm epigenome and the DMR epimutations were not predominant in specific cellular pathways. A gene network analysis of the DMR associated genes did not identify a direct gene connection gene network, but did identify connections with general cellular process, Figure 7.

**Table 1:** Sperm differential methylation regions (DMR) in F3 generation dioxin lineage.

Gene Symbol	Chr	Start	Stop	Gene ID	min p-value	Gene Title
**Cytoskeleton-ECM**						
Flg	2	186309317	186310200	24641	8.5E-15	Filaggrin
**Development**						
Npc2	6	108814526	108815306	286898	3.6E-15	Niemann-Pick disease, type C2
Sema3b	8	112851422	112852727	363142	3.9E-11	sema domain, immunoglobulin domain (Ig), short basic domain, secreted, (semaphorin) 3B
**Epigenetics**						
Jmjd8	10	15093529	15094314	360498	6.1E-07	jumonji domain containing 8
Hdac3	18	30875498	30876873	84578	1.9E-08	histone deacetylase 3
**Golgi Apparatus**						
B4galt2	5	138346044	138347049	313536	1.5E-13	UDP-Gal:betaGlcNAc beta 1,4- galactosyltransferase, polypeptide 2
**Growth Factors**						
Tgfbi	17	13934717	13935412	116487	5.9E-10	transforming growth factor, beta induced
**Hormone**						
LHB	1	95892653	95894255	25329	1.4E-07	luteinizing hormone beta
**Immune Response**						
Irgc1	1	79680291	79680891	308428	8.4E-07	immunity-related GTPase family, cinema 1
Siglec5	1	93734203	93734913	292843	1.4E-09	sialic acid binding Ig-like lectin 5
Fcgr2a	13	86914892	86915576	116591	3.1E-11	Fc fragment of IgG, low affinity IIa, receptor (CD32)
Cd99l2	15	5672856	5673836	171485	9.1E-09	CD99 molecule-like 2
**Metabolism & Transport**						
Syt3	1	94866199	94867099	25731	3.2E-07	synaptotagmin III
Ca2	2	88092498	88093184	54231	2.1E-24	carbonic anhydrase II
Loxl3	4	117244180	117245277	312478	4.2E-11	lysyl oxidase-like 3
Clcn2	11	82429579	82430269	29232	2.7E-08	chloride channel 2
Aldh7a1	18	52310889	52311983	291450	4.9E-17	aldehyde dehydrogenase 7 family, member A1
**Proteolysis**						
DPP3	3	138285960	138287265	114591	8.4E-13	dipeptidylpeptidase 3
Pi16	20	7642807	7643887	294312	2.9E-11	peptidase inhibitor 16
**Receptors & Binding Proteins**						
Olr60	1	160632243	160632843	405017	1.1E-10	olfactory receptor 60
Chrm3	17	71070835	71071435	24260	9.1E-22	cholinergic receptor, muscarinic 3
**Signaling**						
Ppp1r14a	1	84421173	84422179	114004	8.6E-12	protein phosphatase 1, regulatory (inhibitor) subunit 14A
Ffar2	1	85881877	85882477	292794	4.3E-14	free fatty acid receptor 2
Bcar3	2	219075668	219076268	310838	1.1E-14	breast cancer anti-estrogen resistance 3
Dok1	4	117244180	117245277	312477	4.2E-11	docking protein 1
Akap6	6	73042917	73043604	64553	9.5E-85	A kinase (PRKA) anchor protein 6
CSNK1G2	7	10588530	10589932	65278	7.4E-12	casein kinase 1, gamma 2
Shc2	7	11584014	11584614	314612	3.5E-12	SHC (Src homology 2 domain containing) transforming protein 2
Rasal3	7	12968011	12968901	314596	4.2E-11	RAS protein activator like 3
Hspd1	9	53896237	53896837	63868	4.7E-10	heat shock protein 1 (chaperonin)
Grid2ip	12	11553996	11554716	288484	2.4E-08	glutamate receptor, ionotropic, delta 2 (Grid2) interacting protein
Grk6	17	15237197	15237797	59076	1.7E-13	G protein-coupled receptor kinase 6
**Transcription**						
Fes	1	136208036	136209136	361597	4.3E-08	feline sarcoma oncogene
Nras	2	198291944	198293429	24605	9.9E-13	neuroblastoma ras oncogene
Pole3	5	79520269	79520987	298098	5.3E-24	polymerase (DNA directed), epsilon 3 (p17 subunit)
Tceb1	5	1975423	1976200	64525	7.7E-35	transcription elongation factor B (SIII), polypeptide 1
RGD1563216	6	108814526	108815306	500694	3.6E-15	similar to HESB like domain containing 1
Nol10	6	41121100	41121700	313981	3.1E-12	nucleolar protein 10
**Translation & Protein Modification**						
Rpl8	7	114953948	114955044	26962	2.3E-09	ribosomal protein L8
Syncrip	8	93822110	93822710	363113	1.1E-10	synaptotagmin binding, cytoplasmic RNA interacting protein
Padi4	5	159616539	159617242	29512	3.6E-16	peptidyl arginine deiminase, type IV
Rpl36	Un	25163917	25164597	58927	6.0E-20	ribosomal protein L36
Arl6ip4	12	33604987	33605992	65105	2.2E-07	ADP-ribosylation-like factor 6 interacting protein 4
Rpl35	3	18794594	18795299	296709	8.2E-50	ribosomal protein L35
**Miscellaneous & Unknown**						
RGD1307797	1	85716944	85717628	361547	8.9E-08	LOC361547
RGD1560846	12	6095843	6096542	498133	1.5E-11	similar to hypothetical protein MGC40178
NSCAN pred chr14.352.a	14	49589568	49590168		4.2E-12	
NSCAN pred chr14.357.a	14	49806934	49807635		3.5E-10	
Fam129c	16	18796064	18796973	498604	8.8E-09	family with sequence similarity 129, member C
NSCAN pred chr17.082.a	17	12540665	12541742		3.6E-13	

## Discussion

The current study demonstrates that dioxin (TCDD), an environmental toxicant and contaminant present in herbicides such as Agent Orange, can promote epigenetic transgenerational inheritance of diseases in unexposed progeny of the F0 generation females exposed during gestation. These observations extend previous studies in mice [31] and rats [5] by examining a variety of different disease states in 1-year-old rats. In addition, the epigenetic mechanism of transmission of this adult-onset disease was further examined by characterizing the transgenerational epigenetic changes in the F3 generation sperm. Epigenetic alterations in sperm DNA methylation (termed epimutations) in the F3 generation were observed after dioxin exposure of the F0 generation gestating female ancestors. This transgenerational transmission of adult onset diseases has implications of disease risk for not only the current exposed human and animal populations, but also for future generations [1]. For example, Vietnam War Veterans exposed to the Agent Orange descendants may currently be experiencing an increased incidence of disease [7]. The toxic effects of direct exposure to dioxin include acute liver damage, weight loss, thymic atrophy, immune suppression and chronic diseases, as well as lymphomas and leukemias in humans [6]. The list of diseases seen following exposure of war veterans to Agent Orange (herbicide contaminated with dioxin) during the Vietnam era is growing [7]. Similar observations have been made with the Taiwan [11], Seveso Italy [9], China [10] and Japan exposures [33].

Due to the bioaccumulation of dioxin and up to decade long half-life in humans, any woman becoming pregnant even 20 years after dioxin exposure runs the risk of transmitting dioxin effects to her fetus and later generations. A generational study in the Seveso Italy exposed population supports this concept demonstrating health effects in progeny born 25 years following the exposure [13]. Few studies have addressed this transgenerational aspect of dioxin exposure. The first animal study demonstrated transgenerational actions of dioxin on mouse fertility [31]. Subsequently dioxin effects on F3 generation 120 day old rat disease was demonstrated [5]. The current study investigated the adult onset disease in 1 year old F3 generation offspring of F0 generation ancestors exposed to dioxin.

This study did not use toxic doses of dioxin, but used only pharmacological doses based on 0.1% of the oral LD50 dose for dioxin. Therefore, no major toxic effects of dioxin were observed. However, the dose and route of administration used in the current study does not allow risk assessment of dioxin exposure. The objective of the study was to investigate if exposure to TCDD could promote epigenetic transgenerational inheritance of disease/abnormality phenotypes, and not to assess environmental risk of exposure to dioxin. These observations can now be used in future studies with appropriate modes of administration and doses to design more effective risk assessment analysis. However, the current study demonstrates the potential of dioxin to promote epigenetic transgenerational inheritance of disease.

In the current study, the transgenerational diseases/abnormalities observed includes kidney disease, ovary disease/abnormality, and pubertal abnormalities. Kidney disease incidence was higher in the transgenerational F3 generation dioxin lineage males. Chronic kidney disease in humans is correlated with high dioxin levels [34]. Prenatal TCDD exposure has been shown to augment renal immune complex deposition, glomerulonephritis, and mesangial proliferation [35]. Male rats exposed to TCDD have manifested nephrotoxicity shown by increases in serum creatinine and blood urea nitrogen levels, altered kidney histopathology, and renal oxidative stress [36]. Lactational exposure of mice to TCDD caused hydronephrotic kidney [37]. The current study is the first to report a transgenerational kidney histopathology in unexposed F3 generation male descendents of F0 generation gestating females exposed to dioxin.

As previously observed [22], the ovarian disease/abnormality identified included primordial follicle loss and polycystic ovarian disease in F3 generation dioxin lineage females. Currently the world’s human female population is facing an increased incidence of primary ovarian insufficiency, characterized by primordial follicle reserve loss, and an increased incidence of polycystic ovarian disease, characterized by the presence of anovulatory cysts [38], [39]. Similar to kidney disease, ovary disease phenotypes in the current study also appear to be the outcome of epigenetic transgenerational inheritance mechanisms. In animal studies, effects of dioxin exposure on ovarian function and steroid levels have been demonstrated. Dioxin exposure affects ovarian function [40], [41], [42] and results in reduced ovarian weight and reduced numbers of corpora lutea and follicles [43], [44], [45]. Further, dioxin causes reduced ovulation rate, failure of follicular rupture, morphologic changes in the ovary, and abnormal cyclicity with disruption of the estrous cycle [41], [46], [47], [48], [49], [50], [51], [52], [53]. Dioxin slows follicular maturation [51], [52], [54]. Ovarian tumors were induced by chronic TCDD exposures [55]. A nonmonotonic dioxin dose-related association was found with risk of earlier menopause (loss of primordial follicle pool reserve) in a population of women residing near Seveso, Italy, in 1976, at the time of a chemical plant explosion [56]. In the current study, the diseases of primordial follicle loss and polycystic ovarian disease found in the F3 generation support two previous reports [5], [22] of transgenerational ovarian diseases following ancestral exposure to dioxin. It is important to note that the polycystic ovarian disease was observed at an increased frequency in the transgenerational (F3) generation [22]. Therefore, ancestral exposure of a gestating female to dioxin promotes an altered fetal gonadal development and epigenetic reprogramming of the male germline that then transmits the altered epigenome to subsequent generations to contribute to the development of these ovarian diseases transgenerationally. All cell types and tissues derived from the altered sperm epigenome have cell specific alterations in transcriptomes and epigenomes. Previous observations showed a transgenerational alteration in both the transcriptome and the epigenome of the ovarian granulosa cells from F3 generation rats of the vinclozolin lineage [22]. Epigenetic mechanisms underlie the development of polycystic ovary syndrome in women [57] and prenatally androgenized rhesus monkeys [58]. These observations suggest an additional epigenetic paradigm be considered for the etiology of primary ovarian insufficiency and polycystic ovarian disease in women.

Pubertal abnormalities were increased only in female F3 generation animals of dioxin lineage. In an earlier study [5] it was shown that F3 dioxin lineage females had an alteration of the time of pubertal onset (number of days to pubertal onset reduced). The current study assessed the number of animals with pubertal abnormalities using a time of puberty cutoff of mean of control lineage ±2 standard deviations and reports an increased percent incidence of pubertal abnormalities in F3 generation dioxin lineage females. The current study investigated pubertal abnormalities in part due to the dramatic increase in pubertal abnormalities over the past decades in humans [59]. The early and delayed onset of puberty are forerunners to different adult health consequences. For example, early onset of puberty results in accelerated bone mineralization and reduced adult height in girls, as well as susceptibility to breast tumors [60]. The delayed onset of puberty leads to reduced bone mineralization, psychological stress and metabolic problems [60]. In the current study, equal proportions of F1 generation females of dioxin lineage had early and delayed pubertal onset, while males had increased proportion of delayed pubertal onset, indicating sexually dimorphic and different direct-exposure effects. Previously, perinatal exposure to a low dose of dioxin induced only precocious puberty that included early maturation of the hypothalamic-pituitary axis, the gonads and genitals, in female Long-Evans hooded rats [61]. In the current study, the affected F3 generation dioxin lineage females had early onset of puberty, while the affected males showed delayed onset of puberty, indicating sex-specific and different transgenerational effects. In this study pubertal onset in dioxin lineage rats is an example where direct and transgenerational effects are very different. Previously, early onset of puberty in girls has been suggested to be due to environmental exposures to endocrine disruptors [62]. Dioxin exposure is suggested to cause early onset of menarche in girls [63], [64]. Early onset of puberty in girls disrupts brain development, endocrine organ systems and growth leading to susceptibility to disease. It is interesting to note that puberty onset (an early developmental milestone) in this study is associated with epigenetic transgenerational adult onset ovarian disease in F3 generation females.

The molecular mechanism of epigenetic transgenerational inheritance of phenotypes involves the reprogramming of the germline epigenome during male gonadal sex determination [1], [65]. The modified sperm epigenome (DNA methylation) appears to be permanently reprogrammed similar to an imprinted-like site and is protected from DNA demethylation and reprogramming after fertilization and in the following generations. This allows transgenerational transmission of the modified sperm epimutations to then modify all somatic cell and tissue epigenomes and transcriptomes to promote epigenetic transgenerational inheritance of disease phenotypes. The current study further analyzed the altered sperm epigenome and epimutations induced by ancestral dioxin exposure. Transgenerational alterations in an F3 generation sperm epigenome were initially identified following developmental exposure to vinclozolin [2], [4]. A recent study demonstrated a variety of different environmental toxicants induce exposure specific differentially DNA methylated regions (DMR), defined as epimutations and epigenetic biomarkers, which included dioxin lineage F3 generation sperm epimutations [5]. The list of DMR associated genes from F3 generation sperm dioxin lineage is presented in Table S4. Therefore, the sperm epimutations correlated with the epigenetic transgenerational inheritance of the disease phenotypes documented.

Transgenerational diseases are promoted by many environmental compounds [5]. Vinclozolin exposure resulted in F3 generation testis disease, prostate disease, kidney disease, immune system abnormalities, tumors, uterine hemorrhage during pregnancy and polycystic ovarian disease [2], [29], [32], [66]. Further, changes in the methylation patterns of imprinted genes in sperm of F3 generation male mice were found following vinclozolin exposure [67]. Exposure of F0 generation gestating rats to Bisphenol-A caused decreased fertility in F3 generation males [21]. Environmental factors such as nutrition [25] also can promote epigenetic transgenerational inheritance of disease phenotypes. Demonstration of epigenetic transgenerational inheritance in worms [27], flies [28], plants [26] and mammals [30], [68], [69] suggest this phenomenon will likely be critical in biology and disease etiology [1]. Together these observations demonstrate that exposure of gestating females to dioxin during gonadal sex determination promotes epigenetic transgenerational inheritance of adult-onset disease including kidney disease, ovary disease/abnormality and pubertal onset abnormalities. The overall increase in total and multiple diseases/abnormalities in F3 generation are also considerable. Associated with the occurrence of these transgenerational diseases are the epigenetic changes in rat sperm DNA. These epimutations may be useful as early stage biomarkers of compound exposure and adult onset disease. Although not designed for risk assessment, these results have implications for the human populations that are exposed to dioxin and are experiencing declines in fertility and increases in adult onset disease, with a potential to transmit them to later generations.

## Materials and Methods

### Animal Studies and Breeding

All experimental protocols for the procedures with rats were pre-approved by the Washington State University Animal Care and Use Committee (IACUC approval # 02568-026). Female and male rats of an outbred strain Hsd:Sprague Dawley®™SD®™ (Harlan) at about 70 and 100 days of age were fed ad lib with a standard rat diet and ad lib tap water for drinking. To obtain time-pregnant females, the female rats in proestrus were pair-mated with male rats. The sperm-positive (day 0) rats were monitored for diestrus and body weight. On days 8 through 14 of gestation [66], the females were administered daily intraperitoneal injections of dioxin (TCDD 100 ng/kg BW/day) or dimethyl sulfoxide (vehicle control). Treatment lineages are designated ‘control’ and ‘dioxin’ (TCDD) lineages. The gestating female rats treated were designated as the F0 generation. The offspring of the F0 generation rats were the F1 generation. Non-littermate females and males aged 70–90 days from F1 generation of control or dioxin lineages were randomly selected and bred to obtain F2 generation offspring. The F2 generation rats were bred to obtain F3 generation offspring. The F1- F3 generation offspring were not directly treated with the dioxin. No sibling or cousin breeding was used to avoid any inbreeding artifacts. The number of animals used is indicated in Tables S2 and S3, for each histopathology examined. The control lineage population was larger than the dioxin lineage due to the lower incidence of disease in the control lineage. The increased number of control lineage animals allowed for an increased ability to detect disease in the control lineage that then allowed for more accurate statistical comparison of the control versus dioxin lineage populations. No alterations in litter size or sex ratios were identified in the F1, F2 or F3 generations for the control or dioxin lineage animals.

### Tissue Harvest and Histology Processing

Rats at 1-year of age were euthanized by CO_2_ inhalation for tissue harvest. Body and organ weights were measured at dissection time. Testis, epididymis, prostate, seminal vesicle, ovaries, uterus and kidney were fixed in Bouin’s solution (Sigma) and 70% ethanol, then processed for paraffin embedding by standard procedures for histopathology examination. Five-micrometer tissue sections were made and were either unstained and used for TUNEL analysis or stained with H & E stain and examined for histopathology. Blood samples were collected at the time of dissection, allowed to clot, centrifuged and serum samples stored for steroid hormone assays.

### Testicular Apoptotic Cells by TUNEL

Testis sections were examined by Terminal deoxynucleotidyl transferase-mediated dUTP nick end labeling (TUNEL) assay (In situ cell death detection kit, Fluorescein, Roche Diagnostics, Mannheim, Germany). Sections were deparaffinized and rehydrated through an alcohol series. They were deproteinized by Proteinase K (20 mg/ml; Invitrogen, Carlsbad, CA), washed with PBS and then 25 µl of the enzyme-label solution mix was applied and incubated at 37°C for 90 min. After PBS washes, slides were mounted and kept at 4°C until examination in a fluorescent microscope in dark field. Both testis sections of each slide were microscopically examined to identify and to count apoptotic germ cells by the bright fluorescence.

### Histopathology Examination and Disease Classification

All histopathology was examined in randomly selected animals by three independent observers. Testis histopathology criteria included the presence of a vacuole, azoospermic atretic seminiferous tubule and ‘other’ abnormalities including sloughed spermatogenic cells in center of the tubule and a lack of a tubule lumen. Prostate histopathology criteria included the presence of vacuoles, atrophic epithelial layer of ducts and hyperplasia of prostatic duct epithelium. Kidney histopathology criteria included reduced size of glomerulus, thickened Bowman’s capsule and the presence of proteinaceous fluid-filled cysts. A cut-off was established to declare a tissue ‘diseased’ based on the mean number of histopathological abnormalities plus two standard deviations from the mean of control tissues by each of the three individual observers. This number was used to classify rats into those with and without testis, prostate or kidney disease/abnormality in each lineage. A rat tissue section was finally declared ‘diseased’ only when at least two of the three observers marked the same tissue section ‘diseased’. The proportion of rats with obesity or tumor development was obtained by accounting those that had these conditions out of all the animals evaluated. The number of animals per litter (litter representation) mean ± SEM used for the control versus dioxin lineage comparisons for each specific disease/abnormality was found not to be statistically different (p>0.05). Therefore, no litter representation differences or litter bias was detected for any of the specific disease/abnormality assessed.

### Ovarian Disease Analysis by Follicle and Cyst Counts

Every 30^th^ section of each pair of ovaries was stained with hematoxylin and eosin and three stained sections (150 µm apart) through the central portion of the ovary with the largest cross sections being evaluated. Ovary sections were assessed for two histopathologies, primordial follicle loss and polycystic ovary disease. Primordial follicle loss was determined by counting the number of primordial follicles per ovary section and averaging across three sections. An animal was scored as having primordial follicle loss if the primordial follicle number was less than that of the control mean minus two standard deviations. Primordial follicles had an oocyte surrounded by a single layer of either squamous or both squamous and cuboidal granulosa cells [70], [71]. Follicles had to be non-atretic and showing an oocyte nucleus in order to be counted. Polycystic ovary histopathology was determined by microscopically counting the number of small cystic structures per section averaged across three sections. A polycystic ovary was defined as having a number of small cysts that was more than the control mean plus two standard deviations. Cysts were defined as fluid-filled structures of a specified size that were not filled with red blood cells and which were not follicular antrum. A single layer of cells may line cysts. Small cysts were 50 to 250 µm in diameter measured from the inner cellular boundary across the longest axis. Percentages of females with primordial follicle loss or polycystic ovarian disease were computed.

### Analysis of Puberty Onset

Onset of puberty was assessed in females by daily examination for vaginal opening from 30 days of age and in males by balano-preputial separation from 35 days of age. For identifying a rat with a pubertal abnormality the mean from all the rats in control lineage evaluated for pubertal onset was computed and its standard deviation calculated. A range of normal pubertal onset was chosen based on the mean ±2 standard deviations. Any rat with a pubertal onset below this range was considered to have had an early pubertal onset and any rat with a pubertal onset above this range was considered to have had a delayed pubertal onset. The proportion of rats with pubertal abnormalities was computed from the total number of rats evaluated.

### Overall Disease/abnormality Incidence

A table of the incidence of individual diseases/abnormalities in rats from each lineage was created and the proportion of individual disease, total disease and multiple disease incidences was computed. For the individual diseases, only those rats that showed a presence of histopathology (plus) or absence of disease (minus) are included in the computation, Supplemental Tables S3 and S3. For the total diseases/abnormalities, a column with total number of diseases for each rat was created and the number of plus signs were added up for each of the rats and the proportion was computed as the number of rats with total disease out of all the listed rats. For the multiple diseases/abnormalities, the proportion was computed as the number of rats with multiple histopathology out of all the listed rats.

### Epididymal Sperm Collection, Sperm Head Purification, DNA Isolation and Methylated DNA Immunoprecipitation (MeDIP)

The epididymis was dissected free of connective tissue, a small cut made to the cauda and placed in 5 ml of F12 culture medium containing 0.1% bovine serum albumin for 10 minutes at 37°C and then kept at 4°C to immobilize the sperm. The epididymal tissue was minced and the released sperm centrifuged at 13, 000×*g* and stored in fresh buffer at −20°C until processed further. Sperm heads were separated from tails through sonication following previously described protocol (without protease inhibitors) [72] and then purified using a series of washes and centrifugations [73] from a total of nine F3 generation rats per lineage (control or dioxin) that were 120 days of age. DNA extraction on the purified sperm heads was performed as previously described [4]. Equal concentrations of DNA from three individual sperm samples were used to produce three DNA pools per lineage and employed for methylated DNA immunoprecipitation (MeDIP). MeDIP was performed as previously described [4], [5].

### MeDIP-Chip and MeDIP-PCR Analysis

The comparative MeDIP-Chip was performed with Roche Nimblegen’s Rat DNA Methylation 3×720 K CpG Island Plus RefSeq Promoter Array, which contains three identical sub-arrays, with 720,000 probes per sub-array, scanning a total of 15,287 promoters (3,880 bp upstream and 970 bp downstream from transcription start site). Probe sizes range from 50–75 bp in length with the median probe spacing of 100 bp. Three different comparative (MeDIP vs. MeDIP) hybridization experiments were performed (3 sub-arrays) for dioxin lineage versus control, with each subarray encompassing DNA samples from 6 animals (3 each from dioxin and control). MeDIP DNA samples from experimental lineages were labeled with Cy3 and MeDIP DNA samples from the control lineage were labeled with Cy5.

The MeDIP = PCR was used to confirm the MeDIP-Chip analysis observations using two genes. The MeDIP genomic DNA was used for a semiquantitative PCR involving 30 cycles and primers specific to the DMR sites. The genes and primers used were:

Hdac3, 3′TGGCGTATTTCTACGACCCC, 5′GGAATGTTTCCGGTGCCTTC and.

Npc2, 3′AGAATGCTTCCACTTGCCGA, 5′CTCACCGCAGTCCTTGAAGT.

The PCR density for control versus dioxin lineage F3 generation sperm MeDIP samples from three different experiments were determined and normalized for fold change. A mean ± SEM was determined and statistical differences assessed with a U-Mann Whitney analysis.

### Bioinformatic and Statistical Analyses of Chip Data

The bioinformatic analysis was performed as previously described [4], [5]. The statistical analysis was performed in pairs of comparative IP hybridizations between dioxin (D) and controls (C) (e.g. D1-C1 and D2-C2; D1-C1 and D3-C3; D2-C2 and D3-C3). In order to assure the reproducibility of the candidates obtained, only the candidates showing significant changes in all of the single paired comparisons were chosen as a having a significant change in DNA methylation between dioxin lineage and control lineage. This is a very stringent approach to select for changes, since it only considers repeated changes in all paired analyses. Clustered Regions of interest were then determined by combining consecutive probes with changed signal within 600 bases of each other, and based on whether their mean M values were positive or negative, with significance P-values less than 10^−5^. The statistically significant differential DNA methylated regions were identified and P-value associated with each region presented. Each region of interest was then annotated for gene and CpG content. This list was further reduced to those regions with an average intensity value exceeding 9.5 (log scale) and a CpG density ≥1 CpG/100 bp.

Associations between genes containing DMR and particular physiologic cellular processes were determined by an automated, unbiased survey of published literature using Pathway Studio™ software (Ariadne, Elsevier Inc., USA). Signaling pathway enrichment with genes containing DMR was determined by querying the library of KEGG pathways (Kyto Encyclopedia of Genes and Genomes, http://www.genome.jp/kegg/pathway.html).

### Statistical Analysis of Rat Organ and Disease Data

For statistical analysis, all the data on body and organ weights were used as input in the program GraphPad© Prism 5 statistical analysis program and t-tests were used to determine if the data from the dioxin lineage differ from those of control lineages. For the number of rats with disease, logistic regression analysis was used to analyze the data (control or dioxin and diseased or unaffected). All treatment differences were considered significant if P value was less than 0.05.

## Supporting Information

Figure S1 Dioxin and transgenerational endocrine effects. **A.** Control and dioxin F3 generation lineage serum testosterone concentrations. Testosterone concentrations (ng/dl) in F3 generation control and dioxin lineage male rats. **B.** Serum estradiol concentrations in proestrus-estrus in F3 generation control and dioxin lineage females. **C.** Serum estradiol concentrations in diestrus in F3 generation control and dioxin lineage females. **D.** Testicular spermatogenic cell apoptosis. Number of apoptotic germ cells normalized to control means in control (open bars) and dioxin (black bars) lineage (**P<0.01).
(PDF)

Table S1 (PDF)

Table S2 (PDF)

Table S3 (PDF)

Table S4 (PDF)
