# Supplementary figures and images for: Dioxin (TCDD) Induces Epigenetic Transgenerational Inheritance of Adult Onset Disease and Sperm Epimutations
Source: PLoS One. 2012 Sep 26;7(9):e46249. doi: 10.1371/journal.pone.0046249 (PMC3458876; doi:10.1371/journal.pone.0046249)

Supplemental Figure 1

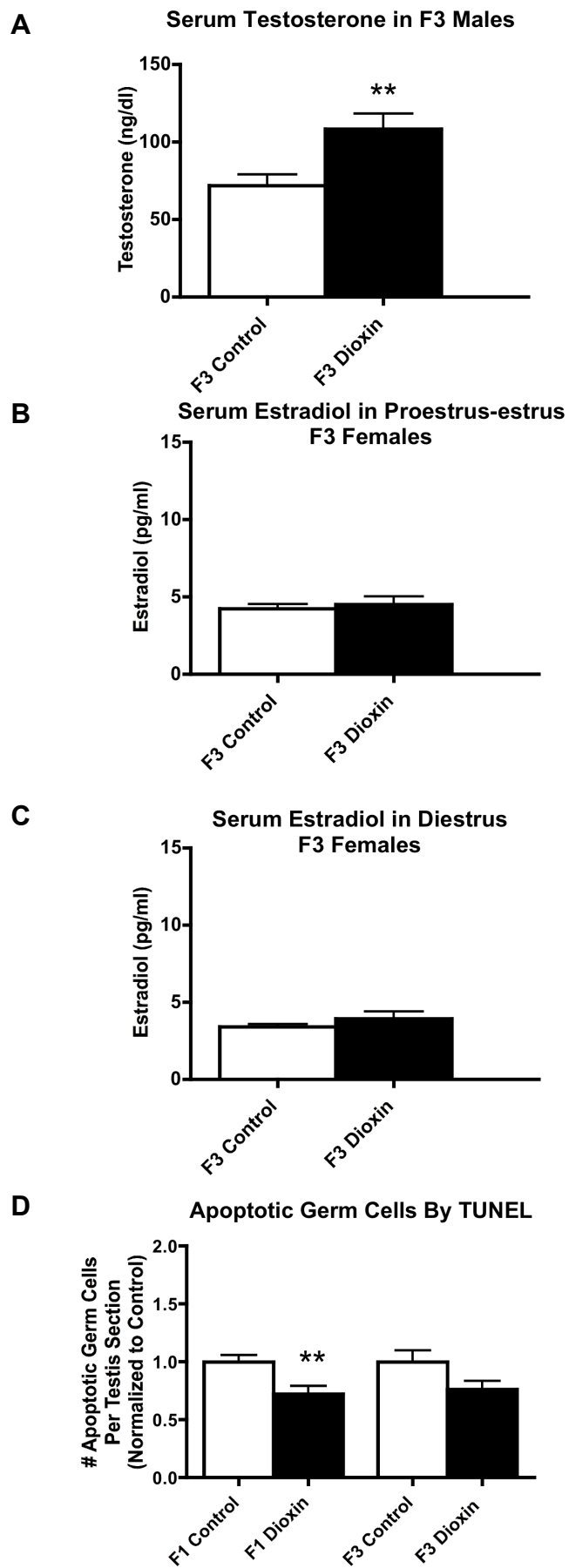

Supplement: Figure S1 — Dioxin and transgenerational endocrine effects. A. Control and dioxin F3 generation lineage serum testosterone concentrations. Testosterone concentrations (ng/dl) in F3 generation control and dioxin lineage male rats. B. Serum estradiol concentrations in proestrus-estrus in F3 generation control and dioxin lineage females. C. Serum estradiol concentrations in diestrus in F3 generation control and dioxin lineage females. D. Testicular spermatogenic cell apoptosis. Number of apoptotic germ cells normalized to control means in control (open bars) and dioxin (black bars) lineage (**P<0.01). (PDF) [file pone.0046249.s001.pdf]
