# Supplementary material for: Dioxin (TCDD) Induces Epigenetic Transgenerational Inheritance of Adult Onset Disease and Sperm Epimutations
Source: PLoS One. 2012 Sep 26;7(9):e46249. doi: 10.1371/journal.pone.0046249 (PMC3458876; doi:10.1371/journal.pone.0046249)
Supplement: Table S1 — (PDF) [file pone.0046249.s002.pdf]

Supplemental Table S1A. Body weight and organ weights in F1 and F3 generation female rats of control and dioxin lineages (mean ± standard error).

Generation	Treatment	Sex	Body Weight (grams)	Ovaries (% of BW)	Uterus (% BW)	Kidney (% BW)
F1	Control	F	310.9	0.0532	0.2589	0.3276
			± 6.15	± 0.0032	± 0.0209	± 0.0127
F1	Dioxin	F	280.2**	0.0485	0.2594	0.3245
			± 6.80	± 0.0048	± 0.0297	± 0.0123
F3	Control	F	293.6	0.0501	0.3883	0.3567
			± 2.31	± 0.0011	± 0.0150	± 0.0036
F3	Dioxin	F	291.6	0.0507	0.3304	0.3405**
			± 3.96	± 0.0018	± 0.0173	± 0.0049

Asterisks (**), if present, indicate statistically significant differences between means of control and dioxin lineage rats ($P<0.01$). The number of animals were as follows: F1 control, 20, F1 dioxin, 9, F3 control 69, F3 dioxin 41.

Supplemental Table S1B. Body weight and organ weights in F1 and F3 generation male rats control and dioxin lineages (mean ± standard error).

Generation	Treatment	Sex	Body Weight (grams)	Testis (% BW)	Prostate (% BW)	Seminal Vesicle (% BW)	Epididymis (% BW)	Kidney (% BW)
F1	Control	M	545.3	0.7599	0.2239	0.1395	0.2585	0.3821
			± 8.66	± 0.0154	± 0.0076	± 0.0092	± 0.0045	± 0.0092
F1	Dioxin	M	527.2	0.8166*	0.1881**	0.1490	0.2675	0.3383**
			± 9.65	± 0.0225	± 0.0076	± 0.0171	± 0.0075	± 0.0068
F3	Control	M	515.80	0.8153	0.1970	0.1330	0.2628	0.3791
			± 6.09	± 0.0102	± 0.0041	± 0.0038	± 0.0028	± 0.0010
F3	Dioxin	M	550.7***	0.7961	0.1890	0.1301	0.2597	0.3419**
			± 6.46	± 0.0116	± 0.0047	± 0.0031	± 0.0035	± 0.0047

Asterisks (*, **, ***), if present, indicate statistically significant differences between means of control and dioxin lineage rats ($P<0.05$, $P<0.01$ and $P<0.001$ respectively). The number of animals were as follows: F1 control 22, F1 dioxin 13, F3 control 56, F3 dioxin 45.
