# Supplementary material for: Dioxin (TCDD) Induces Epigenetic Transgenerational Inheritance of Adult Onset Disease and Sperm Epimutations
Source: PLoS One. 2012 Sep 26;7(9):e46249. doi: 10.1371/journal.pone.0046249 (PMC3458876; doi:10.1371/journal.pone.0046249)
Supplement: Table S2 — (PDF) [file pone.0046249.s003.pdf]

Supplemental Table S2A. Individual disease/abnormality incidence in F1 generation female rats of control and dioxin lineages.

Animal	Animal ID	Sex	Age	Puberty	PFL	PCO	Kidney	Tumor	Obesity	Total Disease
C1	DCS0-1-1-1	F	1 Yr	-	-	-	-	-	-	
C2	DCS0-1-1-2	F	1 Yr	-			-	-	-	
C3	DCS0-1-1-3	F	1 Yr	-			+	-	-	1
C4	DCR1-1-2-1	F	1 Yr	-	-	-	-	-	-	
C5	DCR1-1-2-2	F	1 Yr	-			-	-	-	
C6	DCR1-1-2-3	F	1 Yr	-				+	-	1
C7	DCB1-1-3-1	F	1 Yr	-			-	-	-	
C8	DCB1-1-3-2	F	1 Yr	-				-	-	
C9	DCF1-1-4-1	F	1 Yr	-	-	-	-	+	-	1
C10	DCF1-1-4-2	F	1 Yr	-	-	-	-	-	-	
C11	DCF1-1-4-3	F	1 Yr	-	-	-	+	-	-	1
C12	DCL1-1-5-1	F	1 Yr	-	+	-	+	-	-	2
C13	DCL1-1-5-2	F	1 Yr	-			-	-	-	
C14	DCL1-1-5-3	F	1 Yr	-	-	-	-	-	-	
C15	DCM2-1-6-1	F	1 Yr	-	-	-	-	-	-	
C16	DCM2-1-6-2	F	1 Yr	+			-	-	-	1
C17	DCM2-1-6-3	F	1 Yr	+	-	-	-	-	-	1
C18	DCF0-1-7-1	F	1 Yr	-			-	-	-	
C19	DCF0-1-7-2	F	1 Yr	-			-	-	-	
C20	DCF0-1-7-3	F	1 Yr	-			-	-	-	

Supplemental Table S2A (continued)

Animal	Animal ID	Sex	Age	Puberty	PFL	PCO	Kidney	Tumor	Obesity	Total Disease
H1	DHB0-1-1-2	F	1 Yr	-	+	+	-	-	-	2
H2	DHB0-1-1-3	F	1 Yr	-	+	-	-	-	-	1
H3	DHG2-1-3-2	F	1 Yr	-	+	+	+	-	-	3
H4	DHG2-1-3-3	F	1 Yr	+	+	-	-	-	-	2
H5	DHG2-1-3-4	F	1 Yr	-	+	-	-	-	-	1
H6	DHW1-1-4-2	F	1 Yr	-				-	-	
H7	DHW1-1-4-4	F	1 Yr	-	+	+	-	-	-	2
H8	DHW1-1-4-5	F	1 Yr	-	+	+	-	-	-	2
H9	DHW1-1-4-7	F	1 Yr	-				-	-	

A '+' indicates the presence; A '-' indicates the absence of disease. A blank cell indicates 'not determined.' Animal IDs with a 'C' belong to Control lineage and those with an 'H' belong to Dioxin lineage. PFL = Primordial follicle loss. PCO = Polycystic ovarian disease. See 'Materials and Methods' section for disease assessment in rats. The number of animals per litter (litter representation) mean ± SEM between the control versus dioxin lineage for each specific disease/abnormality was not found to be statistically different ($p>0.05$) for litter representation indicating no litter bias.

Supplemental Table S2B. Individual disease/abnormality incidence in F1 generation male rats of control and dioxin lineages.

Animal	Animal ID	Sex	Age	Puberty	Testis	Prostate	Kidney	Tumor	Obesity	Total Disease
C1	DCS0-1-1-5	M	1 Yr	-	+	-	-	-	-	1
C2	DCS0-1-1-6	M	1 Yr	-	-	-	-	-	-	
C3	DCR1-1-2-5	M	1 Yr	-	-	-	-	-	-	
C4	DCR1-1-2-6	M	1 Yr	-		-	-	-	-	
C5	DCR1-1-2-7	M	1 Yr	-		+	-	-	-	1
C6	DCR1-1-2-8	M	1 Yr	-	-	-	-	-	-	
C7	DCB1-1-3-3	M	1 Yr	-	+	-	-	-	-	1
C8	DCB1-1-3-4	M	1 Yr	-	-	-	-	-	-	
C9	DCF1-1-4-6	M	1 Yr	-	-	-	-	-	-	
C10	DCF1-1-4-7	M	1 Yr	+	-	+	-	-	-	2
C11	DCF1-1-4-8	M	1 Yr	-	-	-	-	-	-	
C12	DCF1-1-4-9	M	1 Yr	+	-	-	-	-	-	1
C13	DCL1-1-5-6	M	1 Yr	-	-	-	-	-	-	
C14	DCL1-1-5-7	M	1 Yr	-	-	+	-	-	-	1
C15	DCL1-1-5-8	M	1 Yr	-	-	-	-	-	-	
C16	DCL1-1-5-9	M	1 Yr	-	-	+	-	-	-	1
C17	DCM2-1-6-7	M	1 Yr	+	-	+	-	-	-	2
C18	DCM2-1-6-9	M	1 Yr	+	+	-	-	-	-	2
C19	DCM2-1-6-10	M	1 Yr	-	+	-	-	-	-	1
C20	DCF0-1-7-5	M	1 Yr	-		-	-	-	-	
C21	DCF0-1-7-6	M	1 Yr	-	-	-	+	-	-	1
C22	DCF0-1-7-7	M	1 Yr	-	-	-	-	-	-	

Supplemental Table S2B (continued)

Animal	Animal ID	Sex	Age	Puberty	Testis	Prostate	Kidney	Tumor	Obesity	Total Disease
H1	DHB0-1-1-4	M	1 Yr	+	-	+		-	-	2
H2	DHB0-1-1-5	M	1 Yr	-	+	+	-	-	-	2
H3	DHB0-1-1-6	M	1 Yr	-	-	+	-	-	-	1
H4	DHB0-1-1-7	M	1 Yr	-	-	+	-	-	-	1
H5	DHB0-1-1-8	M	1 Yr	-	+	+	+	-	-	3
H6	DHB0-1-1-9	M	1 Yr	-	-	+	-	-	-	1
H7	DHK2-1-2-2	M	1 Yr	-	+	-	-	-	-	1
H8	DHK2-1-2-3	M	1 Yr	+	-	-	-	-	-	1
H9	DHK2-1-2-4	M	1 Yr	+	+	+	+		-	4
H10	DHK2-1-2-5	M	1 Yr	+	+	-	+	-	-	3
H11	DHK2-1-2-6	M	1 Yr	+	-	+	-	-	-	2
H12	DHG2-1-3-5	M	10 m	-				+	-	1
H13	DHG2-1-3-6	M	1 Yr	-		+	-	-	-	1

A '+' indicates the presence; A '-' indicates the absence of disease. A blank cell indicates 'not determined.' Animal IDs with a 'C' belong to control lineage and those with an 'H' belong to dioxin lineage. See 'Materials and Methods' section for disease assessment in rats. The number of animals per litter (litter representation) mean ± SEM between the control versus dioxin lineage for each specific disease/abnormality was not found to be statistically different ($p>0.05$) for litter representation indicating no litter bias.
