# Supplementary material for: Dioxin (TCDD) Induces Epigenetic Transgenerational Inheritance of Adult Onset Disease and Sperm Epimutations
Source: PLoS One. 2012 Sep 26;7(9):e46249. doi: 10.1371/journal.pone.0046249 (PMC3458876; doi:10.1371/journal.pone.0046249)
Supplement: Table S3 — (PDF) [file pone.0046249.s004.pdf]

Supplemental Table S3A. Individual disease/abnormality incidence in F3 generation female rats of control and dioxin lineages.

Animal	Animal ID	Sex	Age	Puberty	PFL	PCO	Kidney	Tumor	Obesity	Total Disease
C1	DCF1-3-1-2	F	1 Yr	-			-	-	-	
C2	DCF1-3-1-3	F	1 Yr	-			-	-	-	
C3	DCF1-3-1-4	F	1 Yr	-			-	-	-	
C4	DCF1-3-1-5	F	1 Yr	-				-	-	
C5	DCS0-3-2-2	F	1 Yr	-			-	-	-	
C6	DCS0-3-2-3	F	1 Yr	-	-	-	-	-	-	
C7	DCS0-3-2-4	F	1 Yr	-				-	-	
C8	DCS0-3-2-5	F	1 Yr	-				-	-	
C9	DCL1-3-3-2	F	1 Yr	-	-	-	+	-	-	1
C10	DCL1-3-3-3	F	1 Yr	-				+	-	1
C11	DCB1-3-4-2	F	1 Yr	-			-	-	-	
C12	DCB1-3-4-3	F	1 Yr	-			-	-	-	
C13	DCB1-3-4-4	F	1 Yr	-			-	-	-	
C14	DCB1-3-4-5	F	1 Yr	-	-	-		-	-	
C15	DCB1-3-4-6	F	1 Yr	-				-	-	
C16	DCS0-3-5-2	F	1 Yr	-			+	-	-	1
C17	DCS0-3-5-3	F	1 Yr	-			-	-	-	
C18	DCS0-3-5-4	F	1 Yr	-			-	-	-	
C19	DCS0-3-5-5	F	1 Yr	-			-	-	-	
C20	DCS0-3-5-6	F	1 Yr	-	-	-		-	-	
C21	DCS0-3-6-2	F	1 Yr	-			+	-	-	1
C22	DCS0-3-6-3	F	1 Yr	-	+	-	-	-	-	1
C23	DCB1-3-7-2	F	1 Yr	-	-	-		-	-	

Supplemental Table S3A (continued)

Animal	Animal ID	Sex	Age	Puberty	PFL	PCO	Kidney	Tumor	Obesity	Total Disease
C24	DCB1-3-7-3	F	1 Yr	-			-	-	-	
C25	DCB1-3-7-4	F	1 Yr	-				-	-	
C26	DCB1-3-7-5	F	1 Yr	-				-	-	
C27	DCB1-3-7-6	F	1 Yr	-				-	-	
C28	DCR1-3-8-2	F	1 Yr	-	-	-		-	-	
C29	DCR1-3-8-3	F	1 Yr	-			-	-	-	
C30	DCR1-3-8-4	F	1 Yr	-			-	-	-	
C31	DCR1-3-8-5	F	1 Yr	-				-	-	
C32	DCF1-3-9-2	F	1 Yr	-	-	-		-	-	
C33	DCF1-3-9-3	F	1 Yr	-			-	-	-	
C34	DCF1-3-9-4	F	1 Yr	+			-	-	-	1
C35	DCF1-3-9-5	F	1 Yr					-	-	
C36	DCB1-3-10-2	F	1 Yr	-				-	-	
C37	DCB1-3-10-3	F	1 Yr	-	-	-	-	-	-	
C38	DCB1-3-10-4	F	1 Yr	-			-	-	-	
C39	DCL1-3-11-2	F	10 m.	-				+	-	1
C40	DCL1-3-11-3	F	1 Yr	-			-	-	-	
C41	DCL1-3-11-4	F	1 Yr	-			-	-	-	
C42	DCL1-3-11-5	F	1 Yr	-			-	-	-	
C43	DCL1-3-11-6	F	1 Yr	-				-	-	
C44	DCL1-3-11-8	F	11 m.	-				+	-	1
C45	DCB1-3-12-2	F	1 Yr	-				-	-	
C46	DCB1-3-12-3	F	1 Yr	-			-	-	-	
C47	DCL1-3-13-1	F	1 Yr	-			-	-	-	
C48	DCL1-3-13-2	F	1 Yr	-				-	-	

Supplemental Table S3A (continued)

Animal	Animal ID	Sex	Age	Puberty	PFL	PCO	Kidney	Tumor	Obesity	Total Disease
C49	DCL1-3-13-3	F	1 Yr	-			-	-	-	
C50	DCS0-3-14-2	F	1 Yr	-				-	-	
C51	DCS0-3-14-3	F	1 Yr	-			-	-	-	
C52	DCL1-3-15-2	F	1 Yr	+			-	-	-	1
C53	DCL1-3-15-3	F	1 Yr	+			+	-	-	2
C54	DCL1-3-15-4	F	1 Yr	+			+	-	-	2
C55	DCL1-3-15-5	F	1 Yr	-				-	-	
C56	DCS0-3-16-2	F	1 Yr	-				-	-	
C57	DCS0-3-16-3	F	1 Yr	-			-	-	-	
C58	DCS0-3-16-4	F	1 Yr	-			-	-	-	
C59	DCS0-3-16-5	F	1 Yr	-				-	-	
C60	DCR1-3-17-1	F	1 Yr	-				-	-	
C61	DCR1-3-17-2	F	1 Yr	-			-	-	-	
C62	DCR1-3-17-3	F	1 Yr	-			-	-	-	
C63	DCR1-3-17-4	F	1 Yr	+				-	-	1
C64	DCS0-3-18-2	F	1 Yr					-	-	
C65	DCS0-3-18-3	F	1 Yr				-	-	-	
C66	DCS0-3-18-4	F	1 Yr				-	-	-	
C67	DCF1-3-19-1	F	1 Yr	-				-	-	
C68	DCF1-3-19-2	F	1 Yr	-			-	-	-	
C69	DCF1-3-19-3	F	11 m.	-				+	-	

Animal	Animal ID	Sex	Age	Puberty	PFL	PCO	Kidney	Tumor	Obesity	Total Disease
H1	DHG2-3-1-3	F	1 Yr	-	+	+	-	-	-	2
H2	DHB0-3-2-3	F	1 Yr	-			-	-	-	
H3	DHB0-3-2-4	F	1 Yr	-	+	+	-	-	-	2
H4	DHB0-3-2-5	F	1 Yr	-			-	-	-	
H5	DHG2-3-3-3	F	1 Yr	-			-	-	-	
H6	DHG2-3-3-4	F	1 Yr	-	+	+	-	-	-	2
H7	DHG2-3-3-5	F	1 Yr	-			-	-	-	
H8	DHW1-3-4-3	F	1 Yr	-	+	+	-	-	-	2
H9	DHW1-3-5-3	F	1 Yr	-			-	-	-	
H10	DHW1-3-5-4	F	1 Yr	-	-	+	-	-	-	1
H11	DHW1-3-5-5	F	1 Yr	+			+	-	-	2
H12	DHB0-3-6-2	F	1 Yr	+	-	+	-	-	-	2
H13	DHB0-3-6-3	F	1 Yr	+			-	-	-	1
H14	DHG2-3-7-2	F	1 Yr	-	+	+	-	-	-	2
H15	DHK2-3-8-3	F	1 Yr	-	+	+	+	-	-	3
H16	DHK2-3-8-4	F	1 Yr	-			-	+	-	1
H17	DHK2-3-8-5	F	1 Yr	+			-	-	-	1
H18	DHW1-3-9-3	F	1 Yr	+			+	-	-	2
H19	DHW1-3-9-4	F	1 Yr	+	-	+	+	-	-	3
H20	DHW1-3-9-5	F	1 Yr	-			-	-	-	
H21	DHW1-3-9-6	F	1 Yr	-				-	-	
H22	DHG2-3-10-1	F	1 Yr	+			-	-	-	1
H23	DHG2-3-10-2	F	1 Yr	+			-	-	-	1
H24	DHK2-3-11-1	F	1 Yr	+			-	-	-	1
H25	DHK2-3-11-2	F	1 Yr	+			-	-	-	1
H26	DHK2-3-11-3	F	1 Yr	+			-	-	-	1

Animal	Animal ID	Sex	Age	Puberty	PFL	PCO	Kidney	Tumor	Obesity	Total Disease
H27	DHK2-3-11-4	F	10 m.	+				+	-	2
H28	DHK2-3-11-5	F	1 Yr	+				-	-	1
H29	DHK2-3-11-6	F	1 Yr	+				-	-	1
H30	DHB0-3-12-1	F	1 Yr	-			-	-	-	
H31	DHB0-3-12-2	F	1 Yr	-			+	-	-	1
H32	DHB0-3-12-3	F	1 Yr	-			-	-		
H33	DHW1-3-13-1	F	1 Yr	+			-	-		1
H34	DHW1-3-13-2	F	10 m.	+			-	-		1
H35	DHW1-3-13-3	F	1 Yr	+				-		1
H36	DHW1-3-13-4	F	1 Yr	+			+	-		2
H37	DHW1-3-13-6	F	1 Yr	+				-		1
H38	DHG2-3-14-1	F	1 Yr	+			-	-		1
H39	DHG2-3-14-2	F	1 Yr	+			-	-		1
H40	DHG2-3-14-3	F	1 Yr	+			-	-		1
H41	DHG2-3-14-5	F	1 Yr	+				+		2

A '+' indicates the presence and a '-' indicates the absence of disease. Animal IDs with a 'C' belong to control lineage and those with an 'H' belong to dioxin lineage. PFL = Primordial follicle loss; PCO = Polycystic ovarian disease. See 'Materials and Methods' section for disease assessment in rats. The number of animals per litter (litter representation) mean ± SEM between the control versus dioxin lineage for each specific disease/abnormality was not found to be statistically different ($p>0.05$) for litter representation indicating no litter bias.

Supplemental Table S3B. Individual disease/abnormality incidence in F3 generation male rats of control and dioxin lineages.

Animal	Animal ID	Sex	Age	Pubertal	Testis	Prostate	Kidney	Tumor	Obesity	Total Disease
C1	DCF1-3-1-8	M	1 Yr	-	+		-	-	-	1
C2	DCF1-3-1-9	M	1 Yr	-		-	-	-	-	
C3	DCF1-3-1-10	M	1 Yr	-		-	+	-	-	1
C4	DCS0-3-2-11	M	1 Yr	-	-	-	-	-	-	
C5	DCS0-3-2-12	M	1 Yr	-	-	-	-	-	-	
C6	DCS0-3-2-13	M	1 Yr	-	-		-	-	-	
C7	DCL1-3-3-6	M	1 Yr	-	-	-		-	-	
C8	DCL1-3-3-7	M	1 Yr	-	-	-		-	-	
C9	DCB1-3-4-9	M	1 Yr	-	-	+	-	-	-	1
C10	DCB1-3-4-10	M	1 Yr	-	-	-	-	-	-	
C11	DCB1-3-4-11	M	1 Yr	-	-	-	-	-	-	
C12	DCB1-3-4-12	M	1 Yr	-				-	-	
C13	DCB1-3-4-13	M	1 Yr	-				-	-	
C14	DCS0-3-5-9	M	1 Yr	-	-		-	-	-	
C15	DCS0-3-5-10	M	1 Yr	-	-	-	-	-	-	
C16	DCS0-3-5-11	M	1 Yr	-	-	-	-	-	-	
C17	DCS0-3-6-6	M	1 Yr	-	-		-	-	-	
C18	DCS0-3-6-7	M	1 Yr	-	-	-	-	-	-	
C19	DCS0-3-6-8	M	1 Yr	-	+	-	-	-	-	1
C20	DCB1-3-7-9	M	1 Yr	-	-		+	-	-	1
C21	DCB1-3-7-10	M	1 Yr	-	-	-	-	-	-	
C22	DCB1-3-7-11	M	1 Yr	-	-	-	-	-	-	
C23	DCR1-3-8-7	M	1 Yr	-	-	-	-	-	-	
C24	DCR1-3-8-8	M	1 Yr	-	-	-	-	-	-	

Supplemental Table S3B (continued)

Animal	Animal ID	Sex	Age	Pubertal	Testis	Prostate	Kidney	Tumor	Obesity	Total Disease
C25	DCF1-3-9-11	M	1 Yr	-	-	-		-	-	
C26	DCF1-3-9-12	M	1 Yr	-	-			-	-	
C27	DCF1-3-9-13	M	1 Yr	+	-			-	-	1
C28	DCB1-3-10-9	M	9 m.	-	-	-	-	+	-	1
C29	DCB1-3-10-10	M	1 Yr	-		-	-	-	-	
C30	DCB1-3-10-11	M	1 Yr	-	-	-	-	-	-	
C31	DCB1-3-10-12	M	1 Yr	-	+	-	-	-	-	1
C32	DCB1-3-10-13	M	11m.	-		-		+	-	1
C33	DCL1-3-11-9	M	1 Yr	-	-		-	-	-	
C34	DCL1-3-11-10	M	1 Yr	-	-	+	-	-	-	1
C35	DCL1-3-11-11	M	1 Yr	-	-	-	-	-	-	
C36	DCB1-3-12-6	M	1 Yr	-	-	-	-	-	-	
C37	DCB1-3-12-7	M	1 Yr	-	+	+	-	-	-	2
C38	DCB1-3-12-8	M	1 Yr	-	+	-	-	-	-	1
C39	DCL1-3-13-4	M	1 Yr	-	-	-	-	-	-	
C40	DCS0-3-14-5	M	1 Yr	-	-	-	-	-	-	
C41	DCS0-3-14-6	M	1 Yr	-	-	-		-	-	
C42	DCS0-3-14-7	M	1 Yr	-	-	-	-	-	-	
C43	DCL1-3-15-7	M	1 Yr	+	-	-	-	-	-	1
C44	DCL1-3-15-8	M	1 Yr	+	-	+	-	-	-	2
C45	DCL1-3-15-9	M	1 Yr	+	-	-	-	-	-	1
C46	DCS0-3-16-9	M	1 Yr	-	-	-	-	-	-	
C47	DCS0-3-16-10	M	1 Yr	-	-	-	-	-	-	
C48	DCS0-3-16-11	M	1 Yr	-	-	-	-	-	-	
C49	DCS0-3-16-13	M	1 Yr	-				-	-	

Animal	Animal ID	Sex	Age	Pubertal	Testis	Prostate	Kidney	Tumor	Obesity	Total Disease
C50	DCR1-3-17-8	M	1 Yr	-		-	-	-	-	
C51	DCS0-3-18-10	M	1 Yr			-	+	-	-	1
C52	DCS0-3-18-11	M	1 Yr		+	-	+	-	-	2
C53	DCS0-3-18-12	M	1 Yr		-		-	-	-	
C54	DCF1-3-19-4	M	1 Yr	-	-		-	-	-	
C55	DCF1-3-19-5	M	1 Yr	-	-	-		-	-	
C56	DCF1-3-19-6	M	1 Yr	-	-	-	-	-	-	

Animal	Animal ID	Sex	Age	Pubertal	Testis	Prostate	Kidney	Tumor	Obesity	Total Disease
H1	DHG2-3-1-7	M	1 Yr	-	-		-	-	-	
H2	DHG2-3-1-8	M	1 Yr	-	-	-	-	-	-	
H3	DHG2-3-1-9	M	1 Yr	-	-	-	-	-	-	
H4	DHB0-3-2-14	M	1 Yr	-	-	-	-	-	-	
H5	DHB0-3-2-15	M	1 Yr	+	-	-	-	-	-	1
H6	DHG2-3-3-6	M	1 Yr	-	-	-	+	-	-	1
H7	DHG2-3-3-9	M	1 Yr	-	-	-	+	-	-	1
H8	DHG2-3-3-10	M	1 Yr	-	-	-	+	-	-	1
H9	DHW1-3-4-6	M	1 Yr	-	-	-	-	-	-	
H10	DHW1-3-4-7	M	1 Yr	-		-	-	-	-	
H11	DHW1-3-4-8	M	1 Yr	+	-	-	-	-	-	1
H12	DHW1-3-4-9	M	9 m.	-				-	-	
H13	DHB0-3-6-5	M	1 Yr	-	-	-	-	-	-	
H14	DHB0-3-6-6	M	1 Yr	-	-	-	-	-	-	
H15	DHB0-3-6-7	M	1 Yr	-	-	+	-	-	-	1
H16	DHG2-3-7-4	M	1 Yr	-	-	-	-	-	-	
H17	DHG2-3-7-5	M	1 Yr	-	-	-	+	-	-	1

H18	DHG2-3-7-6	M	1 Yr	-	+	-	-	-	-	1
H19	DHK2-3-8-10	M	1 Yr	-	+	-	-	-	-	1
H20	DHK2-3-8-11	M	1 Yr	-	-	-	+	-	-	1
H21	DHK2-3-8-12	M	1 Yr	-	-	-	-	-	-	
H22	DHW1-3-9-10	M	1 Yr	-	-	-	-	-	-	
H23	DHW1-3-9-11	M	1 Yr	-	-	-	-	-	-	
H24	DHG2-3-10-3	M	1 Yr	-	+	-	-	-	-	1
H25	DHG2-3-10-4	M	1 Yr	-	-	-	-	-	-	
H26	DHG2-3-10-5	M	1 Yr	-	-	-	+	+	-	2
H27	DHG2-3-10-6	M	1 Yr	-				-	-	
H28	DHG2-3-10-7	M	1 Yr	-				-	-	
H29	DHG2-3-10-8	M	1 Yr	-				-	-	
H30	DHK2-3-11-9	M	1 Yr	-	-	-	-	-	-	
H31	DHK2-3-11-10	M	1 Yr	-	+	-	-	-	-	1
H32	DHK2-3-11-11	M	1 Yr	-	+	-	-	-	-	1
H33	DHB0-3-12-5	M	1 Yr	-	+	-	-	-	-	1
H34	DHB0-3-12-6	M	1 Yr	-	+	-	+	-	-	2
H35	DHB0-3-12-7	M	1 Yr	-	-	-	+	-	-	1
H36	DHB0-3-12-8	M	1 Yr	-				-	-	
H37	DHB0-3-12-10	M	1 Yr	-				-	-	
H38	DHW1-3-13-7	M	1 Yr	-	+	-	+	-	-	2
H39	DHW1-3-13-8	M	1 Yr	-	-	-	-	-	-	
H40	DHW1-3-13-9	M	1 Yr	-	-	-	+	-	-	1
H41	DHG2-3-14-6	M	1 Yr	-	-	-	-	-	-	
H42	DHG2-3-14-7	M	1 Yr	-	-	-	-	-	-	
H43	DHG2-3-14-8	M	1 Yr	-	-	-	-	-	-	
H44	DHG2-3-14-9	M	1 Yr	-				-	-	

H45	DHG2-3-14-10	M	1 Yr	-	-	-

A '+' indicates the presence and a '-' indicates the absence of disease. Animal IDs with a 'C' belong to control lineage and those with an 'H' belong to dioxin lineage. See 'Materials and Methods' section for disease assessment in rats. The number of animals per litter (litter representation) mean ± SEM between the control versus dioxin lineage for each specific disease/abnormality was not found to be statistically different ($p>0.05$) for litter representation indicating no litter bias.
