# Supplementary material for: Dioxin (TCDD) Induces Epigenetic Transgenerational Inheritance of Adult Onset Disease and Sperm Epimutations
Source: PLoS One. 2012 Sep 26;7(9):e46249. doi: 10.1371/journal.pone.0046249 (PMC3458876; doi:10.1371/journal.pone.0046249)
Supplement: Table S4 — (PDF) [file pone.0046249.s005.pdf]

Supplementary Table S4. KEGG Pathways enriched with dioxin lineage DMR associated genes.

Pathway Name	# Genes affected	# Genes in Pathway
Ribosome	3	77
Chemokine signaling pathway	3	178
Axon guidance	3	123
Natural killer cell mediated cytotoxicity	3	98
ErbB signaling pathway	2	83
Neuroactive ligand-receptor interaction	2	259
VEGF signaling pathway	2	69
Neurotrophin signaling pathway	2	129
Cholinergic synapse	2	112
Regulation of actin cytoskeleton	2	204
Insulin signaling pathway	2	130
GnRH signaling pathway	2	91
Gastric acid secretion	2	74
Pancreatic secretion	2	103
Tuberculosis	2	69
HTLV-I infection	2	296
Pathways in cancer	2	321
Renal cell carcinoma	2	69
Glioma	2	64
Chronic myeloid leukemia	2	78
